# Planning with care complexity: Factors related to discharge delays of hospitalised people with disability

**DOI:** 10.1111/hsc.13912

**Published:** 2022-07-26

**Authors:** Michele M. Foster, David N. Borg, Vivien Houston, Carolyn Ehrlich, Donna Harre, Geoffrey Lau, Timothy J. Geraghty

**Affiliations:** ^1^ Menzies Health Institute Queensland, The Hopkins Centre Griffith University Brisbane Queensland Australia; ^2^ School of Health Sciences and Social Work Griffith University Brisbane Queensland Australia; ^3^ Division of Rehabilitation Metro South Health Hospital and Health Service Brisbane Queensland Australia

**Keywords:** acquired injury, brain injury, insurance, NDIS, rehabilitation, spinal cord injury

## Abstract

Planning for discharge and supports beyond hospital for people with disability in Australia involves negotiation of complex care systems. The aims of this study were to examine how the individualised support pathway of the National Disability Insurance Scheme (NDIS) functioned for admitted people with disability who required funded support to leave hospital; and to explore the factors indicative of increased care complexity associated with delays. Retrospective chart reviews of people with disability were conducted. Data on 198 eligible patients were extracted, including NDIS plan approval and plan implementation timeframes and discharge delay. Participants’ median age was 52 years (interquartile range = 41–59). The most common disability type was spinal cord injury (41%). The median NDIS plan approval and implementation timeframes were 89 days (63–123) and 39 days (8–131), respectively, and most participants (72%) experienced a delayed discharge. A longer plan implementation timeframe was associated with higher odds of a delay in discharge (OR = 3.41, 95% credible interval = 1.56, 7.11). We did not find any evidence that plan approval timeframe, or any other variable indicative of increased care complexity, was associated with discharge delays. Our findings suggest that a delayed discharge will likely be the reality for people with disability who require funded supports to leave hospital. They also suggest that NDIS plan implementation is a major challenge and a focus for policy and practice improvements. To target solutions, further research should focus on the interactions and negotiations of the multiple intermediaries involved and resource and structural impediments to plan implementation.

**What is known about this topic?**

- Quality and timely planning of supports beyond hospital for people with disability is an ongoing policy and practice challenge in Australia
- Planning typically involves navigation of multiple, disparate complex care systems
- The National Disability Insurance Scheme (NDIS) individualised funded supports promises better planning and supports, although this represents another complex care system

**What this paper adds?**

- An exploration of the dual processes of an individualised support pathway and discharge planning for people with disability in the hospital setting
- Critical scrutiny of how increased system complexity might impact timeframes for planning and discharge
- Future research should focus on the intermediary and resource complexities of implementation of individualised plans for discharge

## 1 INTRODUCTION

For people with disability admitted to hospital in Australia, preparation for discharge and planning of supports beyond hospital has historically involved negotiation between the distinct funding and governance arrangements of health and disability and community support systems. These constitute complex care systems which introduce competing incentives and tensions in the planning process, particularly where there is patient complexity (Redfern et al., 2016). Patient complexity originates from the intersection of personal complications such as long‐term impairments and chronic health needs, and associated planning tasks, as well as the social and environmental factors that influence access to support (Shippee et al., 2012). These patient and system complexities coalesce into a care complexity that involves the interactional effects of multiple agents, and varied tasks and bureaucratic processes, concerned with planning for beyond hospital (Guarinoni et al., 2015; Kuipers et al., 2014). Although arguably the notion of care complexity is not new in healthcare (Guarinoni et al., 2015), the introduction of an individualised support pathway for hospitalised people with disability in Australia, adds another layer of complexity. This raises the potential for interface tensions, which could have varying disruptive effects depending on patient complexities and characteristics of support, and therefore requires critical scrutiny.

The difficulties and disruptions of planning beyond hospital for people with disability are not simply about diagnostic complexity (Bishop & Waring, 2019; Safford et al., 2007). There are often complex behavioural, psychosocial, physical and situational needs to be taken into account (New et al., 2013; Piccenna et al., 2016). Limits on a person's decision‐making capacity can be a further challenge, requiring consideration and involvement of substitute decision‐makers or navigation of guardianship processes (9). There can be a change in housing needs, which necessitates interim care arrangements (Sorensen et al., 2020), and delays locating appropriate housing and supports options (New et al., 2013; Salonga‐Reyes & Scott, 2016). Funding arrangements underpinning support planning can also introduce protracted assessment and approval processes (Sorensen et al., 2020).

The ideal process of managing multiple complexities and planning beyond hospital commences early on in a person's admission, with the goal of coordinated individualised support (Braithwaite et al., 2017; Wong et al., 2011). In Australia, managing care complexities whilst planning beyond hospital for people with disability has recently been assisted by the introduction of the National Disability Insurance Scheme (NDIS) (National Disability Insurance Scheme, 2021a). Individualised funded supports are a centrepiece of the Scheme (National Disability Insurance Scheme, 2021a) to address persistent unmet need and fragmentation in service delivery, are available to Australian citizens if they join the Scheme before 65 years of age and are deemed to have a permanent impairment leading to substantial restrictions in function and participation (Walsh & Johnson, 2013). Although aiming to streamline planning for people with disability from hospital, the NDIS is effectively another complex care system. A report by the Joint Standing Committee on the NDIS transition (Joint Standing Committee on the National Disability Insurance Scheme, 2018), noted that the usual pressures to discharge patients from acute care facilities have been potentially impacted by the operational complexities of the NDIS, necessarily raising concerns about discharge delays. Recently, Houston et al. (Houston et al., 2020) found that the NDIS support planning was associated with delayed discharge for people with acquired brain injury and spinal cord injury, raising concerns about how the complexity of personal and support factors might be impacting the efficiency of transition back to the community. Consequently, this could likely involve disruptions and delays to the hospital discharge process.

### 1.1 An individualised support pathway for hospital discharge

A central feature of the NDIS are funded support packages based on individual needs. Eligibility and access to individualised funded supports is determined through the NDIS pathway, according to what is deemed reasonable and necessary (National Disability Insurance Scheme, 2021b). The complete pathway involves submitting an access request and receiving a response—which included a mandated 21 days for response at the time of the study—and completing a planning meeting, receiving the approved plan, implementing the plan and reviewing outcomes and progress (National Disability Insurance Scheme, 2021b). Hospitalised people with disability who are NDIS participants may require specific supports to enable discharge to the community. In such cases, the steps of access and planning for funded supports often occur in the hospital setting and involve the hospital team engaging the person with disability and interacting with family members, NDIA staff (e.g. NDIS planners) and service providers, to assess functional impairments, understand goals and facilitate the implementation of funded supports for discharge into the community. Supports can include a range of services such as personal care and support workers, transport, modifications to a home, assistive technology (equipment, devices or systems to improve independence), continence aids and other consumables, allied health assessments and therapy. Arguably, this series of planning and decision processes and characteristics of supports mean that being an NDIS participant could add further to care complexity at discharge.

Previous work (Houston et al., 2020) on the NDIS individualised support pathway has focused on the efficiency of pathway processes for hospitalised people with disability, from access request to approved plan, and how this impacted the timing of discharge. Our interest was to understand how increased care complexity, as indicated by decision and support needs, and NDIS planning and implementation timeframes, influenced the transition from hospital to community, in terms of discharge delays. The current study aimed to: (a) describe timeframes for NDIS plan approval and plan implementation (in this study defined as the period between plan approval and hospital discharge) and discharge delays, for people with disability who required funded supports to leave hospital; and (b) explore whether factors indicative of increased care complexity contributed to discharge delays, with a focus on the influence of plan approval and plan implementation timeframes. We hypothesised that: (a) most individuals would experience a discharge delay; and (b) longer plan approval and plan implementation timeframes would be associated with discharge delays.

## 2 MATERIALS AND METHODS

### 2.1 Study overview

Retrospective chart (medical record) reviews of patients with disability who required funded support to leave hospital were conducted. Patients were admitted to one of three hospital facilities in South East Queensland, Australia. Data collection sites, participant eligibility criteria and information on data extraction and variability included in the study are described below.

### 2.2 Data collection sites

Metro South Hospital and Health Service (MSHHS) was purposively selected as an NDIS tracking data system originated within this health service. MSHHS is the most populated hospital and health service in Queensland and a major provider of public health services in Brisbane South, Logan, Redlands and Scenic Rim regions of South East Queensland. MSHHS also manages a demand for funded supports, accommodating hospital facilities with statewide specialist rehabilitation services and acute inpatient addiction and mental health services, and servicing a high proportion of people with disability (Gao et al., 2019). Services are provided via five major hospitals and several health centres. This study included three hospital sites: Princess Alexandra Hospital (a tertiary level facility), Logan Hospital and Redland Hospital. These sites provide care in all adult medical specialities, across acute medical, mental health, cancer, rehabilitation and allied health services, including statewide adult rehabilitation services for newly acquired disability in spinal cord injury (SCI) and acquired brain injury (ABI). Ethical approval for the project was granted by the relevant Human Research Ethics Committee (HREC/2019/QMS/54624).

### 2.3 Data collection

Since 2018, MSHHS has routinely collected administrative data on hospitalised patients who require NDIS‐funded supports to discharge. The data were collected and entered by clinicians at each site into a spreadsheet (known as the NDIS tracker). NDIS tracking data included: patient demographic details and information relating to primary and secondary disabilities; admission, expected date of discharge and actual date of discharge; living situation at admission and discharge; assessed support needs; whether participants had an appointed decision‐maker; dates between NDIS pathway milestones; and clinician documented reasons for delays.

This study used a snapshot of NDIS tracker data from July 1, 2018 to December 31, 2019. This period coincided with the roll‐out of the NDIS in the South‐East corner of Queensland. A member of the research team extracted data from the NDIS tracker for the identified period. A total of 336 participants were identified. Electronic medical records were accessed to extract data which were missing or inaccurate. Participants were included in the study if they met the following criteria: (a) were eligible for the NDIS and were accessing their first plan; (b) required disability‐funded support to leave hospital; and (c) had a minimum data set of assessed support needs available. Applying these criteria, 198 participants were included in the study.

### 2.4 Demographic and disability measures

Demographic variables included: age, gender, marital status and Indigenous status. Age was calculated at hospital admission. Marital status was recorded as: single, married, or de facto, and divorced or separated. Indigenous status was recorded as: neither Aboriginal nor Torres Strait Islander, Aboriginal, Torres Strait Islander or Aboriginal and Torres Strait Islander. Participants' housing situation on admission and discharge was recorded and was used to determine whether there was a change in housing situation at discharge, recorded in a no/yes format. Discharge housing categories included: private residence (owner occupied or rental), social housing, supported accommodation, interim destination (including hospital transfers) and other/unknown.

Participants' primary disability was recorded and categorised as: ABI, amputation, intellectual, neurological, psychosocial and SCI. These six categories were chosen based on NDIS published reports (National Disability Insurance Scheme, 2021c) and in consultation with senior clinicians and researchers working across clinical population groups. ABI included stroke (*n* = 10). Intellectual disabilities included autism (*n* = 1). Neurological disabilities included cerebral palsy (*n* = 1), multiple sclerosis (*n* = 2) and visual impairment (*n* = 1). Data on whether individuals had a secondary disability were recorded, in a no/yes format. Length of stay was calculated as the duration from hospital admission to discharge (in days). Hospital facility type was also noted (i.e. tertiary or non‐tertiary hospital facility).

### 2.5 Measures of care complexity

Participants' potential care complexity was captured in several ways: (a) via five key support needs; (b) whether there was a change in housing situation; (c) whether there was an appointed decision‐maker in place; and (d) through NDIS plan approval and plan implementation timeframes. The five key support needs were: accommodation, assistive technology, behavioural support, home modifications and supported independent living. All participants required personal care support and support co‐ordination, and therefore, these variables were not included in any modelling. NDIS plan approval timeframe was calculated as the number of days between submission of a request to access NDIS and plan approval. In this study, the NDIS plan implementation timeframe was considered the period between date of plan approval and hospital discharge. Although we refer to this period as ‘plan implementation’, it was not possible to determine the extent to which a participant's plan was implemented at their point of discharge from hospital. We expected that longer NDIS plan approval and NDIS plan implementation timeframes would be indicative of a patient with greater care complexity.

### 2.6 Discharge delays

A discharge delay was defined as the period of hospital stay after a patient had been deemed medically fit to leave hospital (Ou et al., 2009; Rojas‐García et al., 2018). In Australian hospitals, an expected date of discharge is determined at the discretion of the treating teams but is often used to provide structure and expectation as to when patients might be ready for discharge (Ou et al., 2009). To determine whether there was a delayed discharge, the number of days between a participant's estimated date of discharge and actual date of discharge was calculated. Zero days would indicate that a participant was discharged on their estimated date of discharge; <0 days would indicate that hospital discharge occurred before the estimated date of discharge; and >0 days would indicate that a participant was discharged after their estimated date of discharge. We considered a delayed discharge as ≥4 days after a participants' estimated date of discharge. This conservative approach accounts for delays of 1–3 days that result from administrative errors, or a change in discharge because of the weekend (i.e. from a Friday to a Monday). If no estimated date of discharge was set, a delay in discharge was noted (*n* = 8), as this often resulted from the treating team being unable to agree on an estimated discharge date due to patient complexity.

Clinician‐documented reasons for a disrupted discharge planning and/or delayed discharge from hospital were also recorded in the NDIS tracker. These open text entries were thematically described as further evidence of reasons for discharge delays.

### 2.7 Data analysis

Descriptive summaries are reported as median and interquartile range (IQR) or count (percent), unless otherwise stated. About 28% of participants were missing NDIS plan approval timeframe data and ~16% were missing NDIS plan implementation timeframe data (Supplement 2). We assumed these data were missing at random, due to the non‐reporting of NDIS milestone dates in electronic records (Borg et al., 2021). Missing values were imputed using predicted mean matching, via the *mice* package (Van Buuren & Groothuis‐Oudshoorn, 2011). The results were averaged over five imputed data sets.

Our primary interest was to investigate how care complexity might contribute to delayed discharge. Bayesian penalised regression was used to determine the covariates that may have contributed to a delayed discharge (Van Erp et al., 2019). Penalised regression guards against overfitting, and in settings where the number of predictors is smaller than the sample size, achieves model parsimony more efficiently compared to traditional variable selection methods (Van Erp et al., 2019). Shrinkage prior distributions (horseshoe [df = 1]) were specified for all covariates in the model, with the aim to shrink small effects to zero, while maintaining true large effects (Carvalho et al., 2009; Piironen & Vehtari, 2017). The ratio of expected non‐zero to zero coefficients was set to 0.2. The model was fit in R (R Core Team, 2021) using Stan (Stan Development Team, 2020) with the *brms* interface (Bürkner, 2017).

Ten predictor variables were included in the model. These variables were chosen because they captured information on participants, their primary disability, the presence of any secondary disabilities and participants' potential care complexity. Predictors were: *age* (standardised; mean = 0, SD = 1), *indigenous status* (levels: non‐Indigenous, Indigenous), *primary disability* (levels: ABI, SCI, other), *secondary disability* (levels: false, true), *change in housing situation* at discharge (levels: false, true), *facility type* (levels: tertiary, non‐tertiary), *NDIS plan approval timeframe* (standardised; mean = 0, SD = 1), *NDIS plan implementation timeframe* (standardised; mean = 0, SD = 1), appointed *decision‐maker* (levels: false, true) and *required supports* (levels: 0, 1, 2, >2). Exploratory plots were generated for all predictors against discharge delay (Supplement 1).

Posterior estimates were generated from Markov chain Monte Carlo procedures (8 chains, each with 10,000 iterations, a 50% burn‐in and thinned by a factor of 5). In interpreting the results, we used estimation methods and focused on conditional effects where there was uncertainty whether there was no effect. This criterion was adopted due to the exploratory nature of the study and the relatively small sample size. As such, our results are likely to be specific to the population for which data were available.

Data are reported as the posterior mean (odds ratio [OR] or marginal probability) and 95% credible interval, unless otherwise stated. We also calculated the probability that an effect was greater than an OR of 1, denoted Pr_OR >1_. The R code can be found at https://github.com/SciBorgo/READY‐study.

## 3 RESULTS

### 3.1 Participants

Participants were generally in their early 50s; half were single, with two in five married or in a de facto relationship (Table 1). Almost half of the samples (48%) were undertaking paid employment at the time of admission. The most common disability type was SCI (41%), followed by ABI (38%). Nearly one in three individuals (31%) were noted as having a second disability. Participants generally discharged to a private residence (79%), with about one in three (39%) experiencing a change in housing situation at discharge compared to admission (Table 1).

**TABLE 1:** Participant demographic characteristics, and income, disability, hospital stay and housing situation variables

Variable	Participants (*n* = 198)
Demographic characteristics	
Age, median (IQR) years	52 (41–59)
Gender, *n* (%)	
Male	136 (69)
Female	62 (31)
Marital status, *n* (%)	
Single	99 (50)
Married/de facto	80 (40)
Divorced/separated	19 (10)
Indigenous status, *n* (%)	
Neither Aboriginal nor Torres Strait Islander	182/197 (92)
Aboriginal and/or Torres Strait Islander	15/197 (8)
Income	
Income source on admission, *n* (%)	
Paid employment	94 (48)
Centrelink payment/pension	70 (35)
Self‐funded/retired	10 (5)
Other/unknown	24 (12)
Disability	
Primary disability type, *n* (%)	
Acquired brain injury	75 (38)
Amputation	14 (7)
Intellectual	3 (2)
Neurological	11 (6)
Psychosocial	13 (7)
Spinal cord injury	82 (41)
Secondary disability, *n* (%)	62 (31)
Hospital	
Delay in hospital discharge, *n* (%)	142 (72)
Length of stay, median (IQR) days^a^	175 (110–294)
NDIS plan approval timeframe, median (IQR) days^b^	89 (63–123)
NDIS plan implementation timeframe, median (IQR) days^c^	39 (8–131)
Tertiary hospital facility, *n* (%)	175 (88)
Housing	
Housing situation at discharge, *n* (%)	
Private residence (includes rental and owner occupied)	156 (79)
Social housing	13 (7)
Cared accommodation	8 (4)
Other	21 (11)
Change in housing situation at discharge compared to admission, *n* (%)	77 (39)
Support needs	
Accommodation	83 (42)
Assistive technology	166 (84)
Behavioural support	38 (19)
Home modifications	99 (50)
Supported independent living	38 (19)

Abbreviations: IQR, interquartile range; NDIS, National Disability Insurance Scheme.

^a^ Length of stay was calculated as the number of days between hospital admission and discharge.

^b^ Calculated as the number of days between access request submission and plan approval.

^c^ Calculated as the number of days between plan approval and hospital discharge.

### 3.2 Required support needs

Required support needs are reported in Table 1. The most common type of required supports was assistive technology (85%), followed by home modifications (50%) and accommodation supports (42%).

Most participants (88%) at a tertiary facility type required assistive technology, and about half (55%) required home modifications (Supplement 3). This is likely explained by the large proportion of participants with ABI (89%) and SCI (99%) who attended a tertiary facility (Supplement 4). Around three in five participants at a non‐tertiary facility required accommodation (61%) and behavioural (57%) supports and supported independent living (57%; Supplement 4).

Across the disability types (Supplement 4), most participants with ABI (83%) and amputation (93%), and all participants with a SCI or intellectual disability required assistive technology. Home modifications were required by about two‐thirds of participants with an amputation (57%) or SCI (65%). Supported independent living was required by all participants with an intellectual disability, and by more than half of those with a neurological (55%) or psychosocial (62%) disability. Unique combinations of required support needs are reported in Supplement 5.

### 3.3 Factors related to discharge delay

**FIGURE 1:**
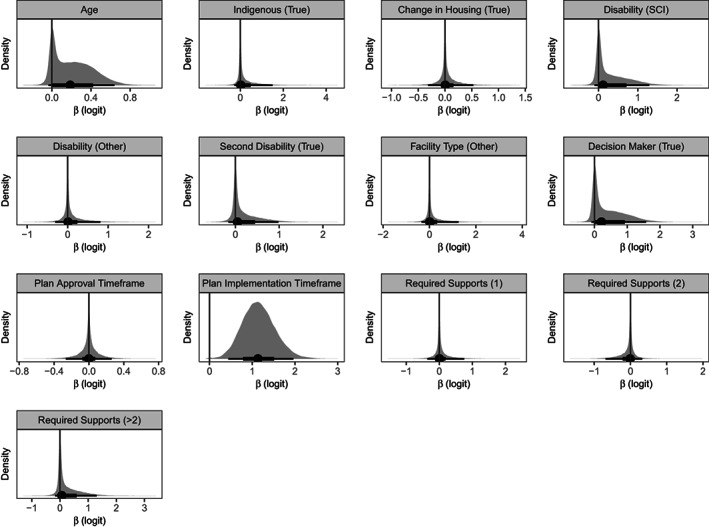
Parameter estimates from the Bayesian penalised regression model predicting discharge delay. The posterior mean (circle) is shown with 66% (thick inner line) and 95% (thin outer line) credible intervals. Grey shaded area indicates the smoothed density distribution of the data, with the height proportional to the number of data points that fall in that part of the distribution. Dark grey line indicates zero. Age, and plan approval and plan implementation timeframe were standardised before analysis. SCI, spinal cord injury.

**FIGURE 2:**
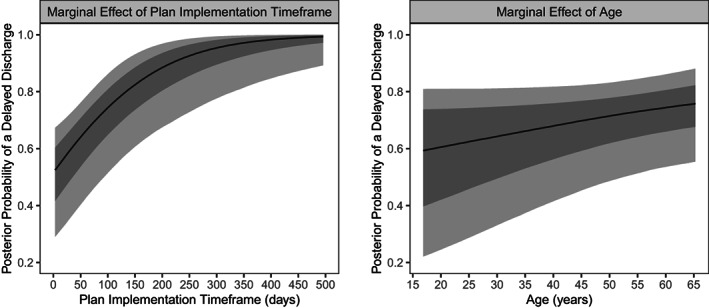
The marginal effects of the National Disability Insurance Scheme (NDIS) plan implementation timeframe (considered the period between NDIS plan approval and hospital discharge) and age on discharge delay. Posterior probabilities are reported as the mean (solid black line) with 66% (inner grey ribbon) and 95% (outer grey ribbon) credible intervals.

Participants with a longer NDIS plan implementation timeframe were more likely to experience a delayed discharge (OR = 3.41, 95% credible interval = 1.56 to 7.11; Pr_OR >1_ = 0.999; Figure 1). There was weak evidence that older participants may have been more likely to experience a discharge delay, to a small extent (OR = 1.26, 95% credible interval = 0.97 to 1.90; Pr_OR >1_ = 0.877; Figure 1). Marginal posterior probabilities of a delay in discharge, as a function of plan implementation timeframe and age are displayed in Figure 2. We did not find any evidence that plan approval timeframe, or any other measures of care complexity, were related to discharge delay (Figure 1). Of note, the proportion of variance in discharge delay explained by the model was low, at 15% (*R* ^2^ = 0.147, 95% credible interval = 0.053–0.248) (Gelman et al., 2019).

### 3.4 Documented reasons for a disrupted discharge planning and/or delayed discharge

Approximately 75% (*n* = 148/198) of the participants included from the NDIS tracking data had comments recorded by the treating team about their NDIS pathway and discharge process, including reasons for discharge delays. In many cases, there was more than one reason provided for a delay to discharge. Reasons for discharge delays related to both the planning and decision about supports, as well as implementation once the plan had been approved. Major reasons were: (1) issues relating to home modifications, which included funding decisions by the NDIA, delays in obtaining professional assessments or a protracted timeframe to progress modifications (*n* = 18); (2) waiting on application for suitable accommodation, or challenges locating appropriate accommodation and/or supported independent living arrangements (*n* = 35); and (3) issues relating to funding and provision of equipment and assistive technology (*n* = 12). Behavioural support needs (*n* = 9) and issues relating to care and support such as locating appropriate providers were other issues complicating discharge planning and the NDIS pathway based on documentation. For some participants, comments indicated that challenges in terms of the NDIS pathway processes and interface with health, for example, relating to communications between the treating team and the agency staff, protracted waiting times for responses from the agency and delays between milestones such as delays in getting a planning meeting.

## 4 DISCUSSION

The current study described the timeframes for the NDIS in hospital pathway and discharge delays for people with disability, who required funded supports to leave hospital. We also investigated how factors related to increased care complexity may have contributed to discharge delays, with a primary interest in the effects of NDIS plan approval and implementation timeframes on discharge delay. As hypothesised, most participants (72%) experienced a delayed discharge, and we found evidence that a longer period between the approval of a plan and discharge was associated with delays (Figure 2). Although we did not find any evidence that any other variable indicative of increased care complexity was related to delays, documentation by clinicians in the NDIS tracker indicated that the types and combination of support needs were disrupting transition and contributing to delays. In this case, waiting on appropriate accommodation or supported independent living may have been contributing to a protracted timeframe between plan approval and hospital discharge. Whilst this finding suggests availability and access are problematic, associated with this is the question of individual preferences and how these might contribute to protracted waiting times.

Previous research on the discharge of hospitalised people with disability has shown that the challenges of finding appropriate accommodation options can delay a timely transition (Redfern et al., 2016), which further supports our clinician documented reasons for delays. Similarly, issues with progressing home modifications and the provision of equipment and assistive technology were also impediments to a timely discharge (National Disability Insurance Scheme, 2021d; Redfern et al., 2016). This is consistent with previous research on medically complex patients, such as people with SCI, which has shown that waiting for long‐term supports and home modifications and provision of equipment contributed to unnecessary hospital stays and transition delays (New et al., 2013).

We found no evidence that period from submission of a request to access NDIS and plan approval (i.e. plan approval timeframe) was related to discharge delay. However, the current study goes further than previous work (Houston et al., 2020), in highlighting plan implementation as a potentially more complicated part of the pathway to discharge. Aside from the availability of supports noted, the actual implementation was likely complicated by the involvement of multiple agents and the many inter‐organisational interactions that transpire (Bishop & Waring, 2019; Braithwaite et al., 2017). As previously argued, where longer term supports are the object of planning, this can exacerbate the usual system complexities and boundary tensions (Bishop & Waring, 2019), particularly where there are debates about what counts as reasonable therapy and support (Zasler et al., 2013).

Quality and timely planning of supports beyond hospital for people with complex needs is a policy and practice challenge (Fuji et al., 2013). In this case, the NDIS implementation was relatively new in the study sites. It is critical, therefore, to map and examine these complex policy interactions and processes to enable understanding of the consequences, the precise dynamics contributing to negative impacts, and to develop targeted improvements (Nevile et al., 2019). To that end, quality data for mapping processes are important, but equally so are the perspectives and experiences of those who implement planning processes—both NDIS and discharge. Frontline professionals and personnel are key to the interpretation of complex policies and are rightly placed to identify interface challenges and where to intervene to avoid adverse impacts and improve good practices (Nevile et al., 2019). Likewise, support coordination was not considered in the model since all participants had support coordination; however, both the quality and complexities of coordination are likely to vary (Foster et al., 2021). Yet professionals, planners and coordinators are critical intermediaries in facilitating planning and discharge. Future research could build on this study by capturing information on the interactions and capabilities of clinical staff (e.g. disability and NDIS related), NDIS planners (e.g. clinical awareness) and other critical intermediaries (De Grood et al., 2016).

Several limitations should be acknowledged. First, we considered a delay in discharge as a transition from hospital to community four or more days after the estimated discharge date. Defining a delay using this criterion may have been too conservative. Seven participants (3.5%) in the current study had a delay of 1–3 days, which we did not consider as a delay. We also noted a delay for eight participants (4%) who did not have an estimated date of discharge. Second, it is possible that we have not captured all variables indicative of care complexity, nor all the barriers associated with a delayed discharge. In support of this statement, our model explained only 15% of the variance in discharge delay. Previous studies in non‐disability populations (Ragavan et al., 2017; Safavi et al., 2019) and therefore, patients with less complex discharges, identified up to ~180 barriers to discharge delay (Ragavan et al., 2017), albeit in American populations. Third, we could not discern from the NDIS tracker data whether participants were discharged with full or partial plan implementation, or before the implementation of supports. Future research should continue to investigate in hospital NDIS pathways, and their effects on discharge delay, in non‐metropolitan hospital facilities, as the factors that impact planning and discharge timeframes are likely to vary across regions.

## 5 CONCLUSION

The most common reality for people with disability who require funded supports to leave hospital is a delayed discharge. Our study shows that NDIS in hospital pathway processes, and specifically the period from plan approval to discharge, may be associated with delays, suggesting that NDIS plan implementation is a major challenge. This warrants attention from both policy and practice. Whilst we did not find evidence that plan approval timeframes were associated with delays, this is not to say that plan approval processes do not, at least in part, contribute to discharge delays. Future studies should take a closer look at the dynamics of implementation once plans are approved. Moreover, future research should examine the impact of in‐hospital NDIS processes on discharge delays across a larger number of facilities to increase the generalisability of our findings and consider regional differences on NDIS pathways and their association with delays in the transition from hospital to community.

## AUTHOR CONTRIBUTIONS

All authors made a significant contribution to this manuscript, including study conception and design, execution, acquisition of data, and data analysis and interpretation. All authors were involved in drafting, revising and critically reviewing the manuscript, and gave approval of the version to be published.

## CONFLICT OF INTEREST

The authors declare that they have no conflicts of interest relevant to the content in this article.

## FUNDING INFORMATION

This project was conducted with grant funding from the Summer Foundation. This support does not represent an endorsement of the contents or conclusions of the manuscript. The funder had no role in the study design, data collection or data analysis, decision to publish or preparation of the manuscript.

## Supporting information


Supplement 1

Supplement 2

Supplement 3

Supplement 4

Supplement 5

## Data Availability

The R code that supports all analyses is available at https://github.com/SciBorgo/READY‐study. A de‐identified data set can be obtained upon reasonable request to the corresponding author.
