# Supplementary material for: Planning with care complexity: Factors related to discharge delays of hospitalised people with disability
Source: Health Soc Care Community. 2022 Jul 26;30(6):e4992–5000. doi: 10.1111/hsc.13912 (PMC10087249; doi:10.1111/hsc.13912)

**Supplement 1.** Exploratory plots of all model predictions against discharge delay. Individual participant data are shown in circles on panels *Age*, *NDIS Plan Approval Timeframe* and N*DIS Plan Implementation Timeframe*. Panels Indigenous Status, *Primary Disability Type*, *Change in Housing at Discharge*, *Secondary Disability*, *Facility Type*, *Appointed Decision Maker* and *Required Supports* are visual representations of contingency tables, with the size of the circles proportional to number of counts (participants) in those cells.


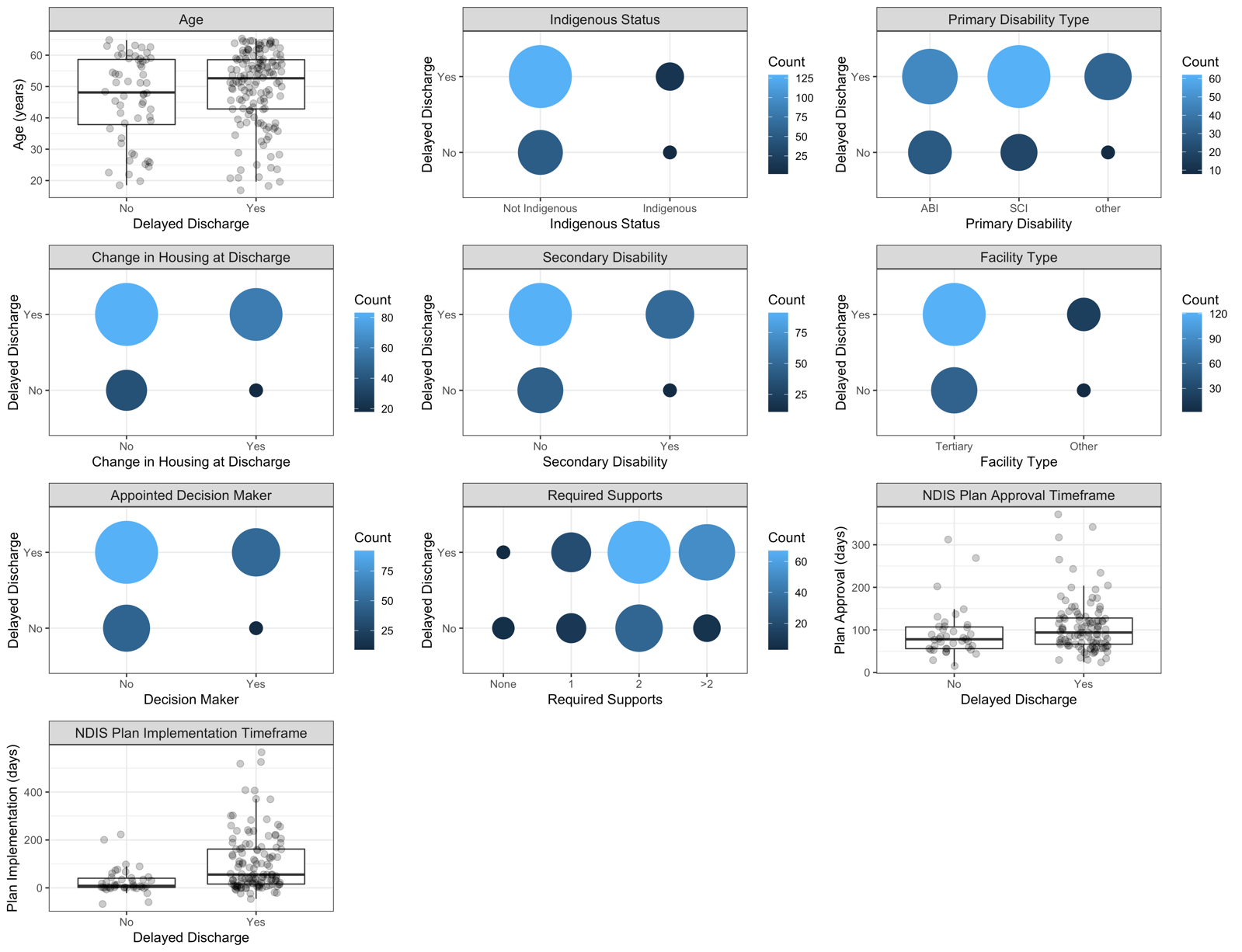

Supplement: Supplementary file 1 — Supplement 1 [file HSC-30-e4992-s003.docx]
