# Supplementary material for: Planning with care complexity: Factors related to discharge delays of hospitalised people with disability
Source: Health Soc Care Community. 2022 Jul 26;30(6):e4992–5000. doi: 10.1111/hsc.13912 (PMC10087249; doi:10.1111/hsc.13912)

**Supplement 2.** Missing data over plots. Panel A shows the percentage of missing data within each variable and overall. Panel B shows missing data as a function of discharge delay. About 28% of plan approval data and 16% of plan implementation data were missing. These values were assumed missing at random.


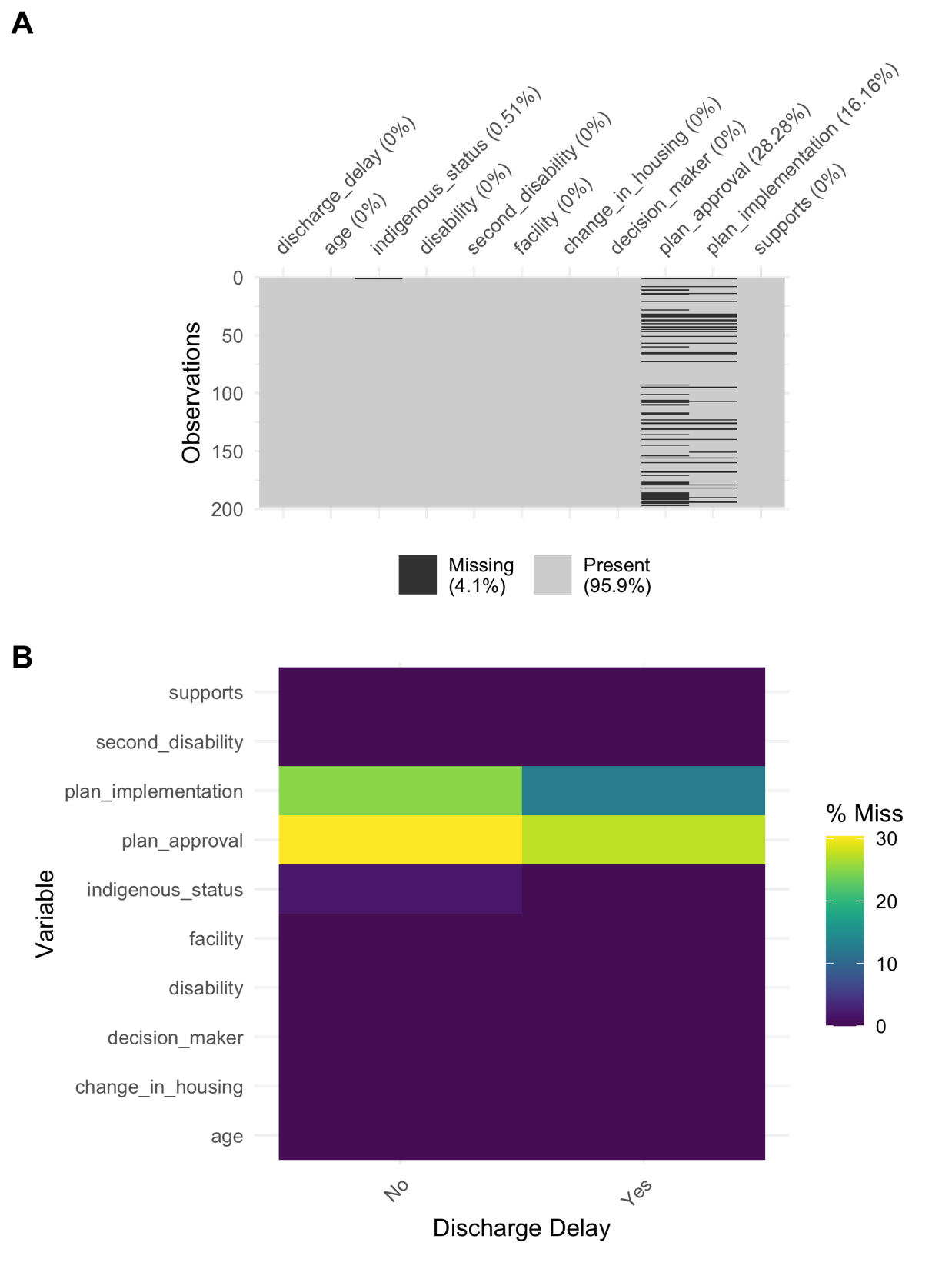

Supplement: Supplementary file 2 — Supplement 2 [file HSC-30-e4992-s004.docx]
