# Supplementary material for: Planning with care complexity: Factors related to discharge delays of hospitalised people with disability
Source: Health Soc Care Community. 2022 Jul 26;30(6):e4992–5000. doi: 10.1111/hsc.13912 (PMC10087249; doi:10.1111/hsc.13912)
Supplement: Supplementary file 3 — Supplement 3 [file HSC-30-e4992-s005.docx]

**Supplement 3.**Participant demographic characteristics, and income, disability, hospital stay and housing situation variables, reported by facility type.

| Variable | Tertiary facility (n=175) | Other facility (n=23) |
| --- | --- | --- |
| ***Demographic characteristics*** |  |  |
| Age, median (IQR) years | 52 (42–59) | 51 (39–56) |
| Gender, n (%) |  |  |
| Male | 123 (70) | 13 (57) |
| Female | 52 (30) | 10 (43) |
| Marital status, n (%) |  |  |
| Single | 83 (47) | 16 (70) |
| Married/de facto | 76 (43) | 4 (17) |
| Divorced/separated | 16 (9) | 3 (13) |
| Indigenous status, n (%) |  |  |
| Neither Aboriginal nor Torres Strait Islander | 162 (93) | 20/22 (91) |
| Aboriginal and/or Torres Strait Islander | 13 (7) | 2/22 (9) |
| ***Income*** |  |  |
| Income source on admission, n (%) |  |  |
| Paid employment | 91 (52) | 3 (13) |
| Centrelink payment/pension | 55 (31) | 15 (65) |
| Self-funded/retired | 10 (6) | 0 (0) |
| Other/unknown | 19 (11) | 5 (22) |
| ***Disability*** |  |  |
| Primary disability type, n (%) |  |  |
| Acquired brain injury | 67 (38) | 8 (35) |
| Amputation | 14 (8) | 0 (0) |
| Intellectual | 2 (1) | 1 (4) |
| Neurological | 6 (3) | 5 (22) |
| Psychosocial | 5 (3) | 8 (35) |
| Spinal cord injury | 81 (46) | 1 (4) |
| Secondary disability, n (%) | 52 (30) | 10 (44) |
| ***Hospital*** |  |  |
| Length of stay, median (IQR) days ^a^ | 166 (105–282) | 272 (207–526) |
| NDIS plan approval timeframe, median (IQR) days ^b^ | 88 (63–122) | 108 (67–128) |
| NDIS plan implementation timeframe, median (IQR) days ^c^ | 36 (7–106) | 150 (53–336) |
| ***Housing*** |  |  |
| Housing situation at discharge, n (%) |  |  |
| Private residence (rental and owner occupied) | 143 (82) | 13 (57) |
| Social housing | 11 (6) | 2 (9) |
| Cared accommodation | 4 (2) | 4 (17) |
| Other | 17 (10) | 4 (17) |
| Change in housing situation at discharge compared to admission, n (%) | 62 (35) | 15 (65) |
| ***Support needs*** |  |  |
| Accommodation | 69 (39) | 14 (61) |
| Assistive technology | 154 (88) | 12 (52) |
| Behavioural support | 25 (14) | 13 (57) |
| Home modifications | 97 (55) | 2 (9) |
| Supported independent living | 25 (14) | 13 (57) |

*Note*. NDIS = National Disability Insurance Scheme, IQR = interquartile range.

^a^ Length of stay was calculated as the number of days between hospital admission and hospital discharge.

^b^ Calculated as the number of days between access request submission and plan approval.

^c^ Calculated as the number of days between plan approval and hospital discharge.
