# Supplementary material for: Planning with care complexity: Factors related to discharge delays of hospitalised people with disability
Source: Health Soc Care Community. 2022 Jul 26;30(6):e4992–5000. doi: 10.1111/hsc.13912 (PMC10087249; doi:10.1111/hsc.13912)
Supplement: Supplementary file 4 — Supplement 4 [file HSC-30-e4992-s001.docx]

**Supplement 4.**Participant demographic characteristics, and income, disability, hospital stay and housing situation variables and required supports, reported by primary disability type.

| Variable | Acquired brain injury (n=75) | Amputation  (n=14) | Intellectual  (n=3) | Neurological  (n=11) | Psychosocial  (n=13) | Spinal cord injury (n=82) |
| --- | --- | --- | --- | --- | --- | --- |
| ***Demographic characteristics*** |  |  |  |  |  |  |
| Age, median (IQR) years | 52 (39–58) | 58 (54–60) | 43 (32–53) | 56 (47–60) | 43 (39–51) | 51 (41–59) |
| Gender, n (%) |  |  |  |  |  |  |
| Male | 52 (69) | 11 (79) | 2 (67) | 8 (73) | 8 (62) | 55 (67) |
| Female | 23 (31) | 3 (21) | 1 (33) | 3 (27) | 5 (39) | 27 (33) |
| Marital status, n (%) |  |  |  |  |  |  |
| Single | 28 (37) | 9 (64) | 2 (67) | 8 (73) | 10 (77) | 42 (51) |
| Married/de facto | 39 (52) | 4 (27) | 0 (0) | 2 (18) | 2 (15) | 33 (40) |
| Divorced/separated | 8 (11) | 1 (7) | 1 (33) | 1 (9) | 1 (8) | 7 (9) |
| Indigenous status, n (%) |  |  |  |  |  |  |
| Neither Aboriginal nor Torres Strait Islander | 74 (99) | 12 (86) | 3 (100) | 11 (100) | 10/12 (83) | 72 (88) |
| Aboriginal and/or Torres Strait Islander | 1 (1) | 2 (14) | 0 (0) | 0 (0) | 2/12 (17) | 10 (12) |
| ***Income*** |  |  |  |  |  |  |
| Income source on admission, n (%) |  |  |  |  |  |  |
| Paid employment | 48 (64) | 2 (14) | 0 (0) | 1 (9) | 1 (8) | 42 (51) |
| Centrelink payment/pension | 15 (20) | 8 (57) | 2 (67) | 6 (55) | 12 (92) | 27 (33) |
| Self-funded/retired | 3 (4) | 1 (7) | 0 (0) | 1 (9) | 0 (0) | 5 (6) |
| Other/unknown | 9 (12) | 3 (21) | 1 (33) | 3 (27) | 0 (0) | 8 (10) |
| ***Disability*** |  |  |  |  |  |  |
| Secondary disability, n (%) | 17 (23) | 3 (21) | 3 (100) | 6 (55) | 8 (62) | 25 (31) |
| ***Hospital*** |  |  |  |  |  |  |
| Length of stay, median (IQR) days ^a^ | 170 (104–288) | 108 (83–136) | 380 (247–476) | 348 (188–562) | 233 (154–711) | 175 (112–274) |
| NDIS plan approval timeframe, median (IQR) days ^b^ | 104 (63–148) | 72 (67–78) | 83 (75–144) | 93 (82–116) | 46 (41–52) | 89 (63–115) |
| NDIS plan implementation timeframe, median (IQR) days ^c^ | 36 (6–98) | 15 (13–26) | 302 (152–355) | 198 (70–342) | 150 (71–254) | 38 (9–106) |
| Tertiary hospital facility, n (%) | 67 (89) | 14 (100) | 2 (67) | 6 (55) | 5 (39) | 81 (99) |
| ***Housing*** |  |  |  |  |  |  |
| Housing situation at discharge, n (%) |  |  |  |  |  |  |
| Private residence (includes rental and owner occupied) | 68 (91) | 13 (93) | 0 (0) | 3 (27) | 6 (46) | 66 (80) |
| Social housing | 2 (3) | 0 (0) | 0 (0) | 1 (9) | 1 (8) | 9 (11) |
| Cared accommodation | 1 (1) | 0 (0) | 0 (0) | 4 (36) | 0 (0) | 1 (1) |
| Other | 4 (5) | 1 (7) | 3 (100) | 3 (27) | 4 (31) | 6 (7) |
| Change in housing situation at discharge vs. admission, n (%) | 29 (39) | 3 (21) | 3 (100) | 7 (64) | 8 (62) | 27 (33) |
| ***Support needs*** |  |  |  |  |  |  |
| Accommodation | 30 (40) | 3 (21) | 3 (100) | 7 (77) | 10 (77) | 30 (37) |
| Assistive technology | 62 (83) | 13 (93) | 3 (100) | 4 (36) | 2 (15) | 82 (100) |
| Behavioural support | 15 (20) | 0 (0) | 2 (67) | 9 (82) | 7 (54) | 5 (6) |
| Home modifications | 35 (47) | 8 (57) | 0 (0) | 3 (27) | 0 (0) | 53 (65) |
| Supported independent living | 20 (27) | 0 (0) | 3 (100) | 6 (55) | 8 (62) | 1 (1) |

*Note*. NDIS = National Disability Insurance Scheme, IQR = interquartile range.

^a^ Length of stay was calculated as the number of days between hospital admission and hospital discharge.

^b^ Calculated as the number of days between access request submission and plan approval.

^c^ Calculated as the number of days between plan approval and hospital discharge.
