# Supplementary material for: Planning with care complexity: Factors related to discharge delays of hospitalised people with disability
Source: Health Soc Care Community. 2022 Jul 26;30(6):e4992–5000. doi: 10.1111/hsc.13912 (PMC10087249; doi:10.1111/hsc.13912)
Supplement: Supplementary file 5 — Supplement 5 [file HSC-30-e4992-s002.docx]

**Supplement 5.** Unique combinations of required support needs.

| Required support, or combinations of required supports | Participants (n=198) | Percent of participants with a discharge delay |
| --- | --- | --- |
| Assistive technology, home modifications | 71 (35.9%) | 63% |
| Assistive technology | 25 (12.6%) | 68% |
| Accommodation, assistive technology | 22 (11.1%) | 68% |
| Accommodation, assistive technology, home modifications | 15 (7.6%) | 80% |
| Accommodation, supported independent living, behavioural support | 12 (6.1%) | 92% |
| Accommodation, supported independent living, assistive technology | 10 (5.1%) | 80% |
| *No supports required* | 8 (4.0%) | 38% |
| Accommodation, supported independent living, assistive technology, behavioural support | 8 (4.0%) | 88% |
| Assistive technology, home modifications, behavioural support | 5 (2.5%) | 80% |
| Accommodation, behavioural support | 3 (1.5%) | 100% |
| Accommodation, assistive technology, behavioural support | 3 (1.5%) | 100% |
| Accommodation, supported independent living | 3 (1.5%) | 100% |
| Behavioural support | 2 (1.0%) | 100% |
| Home modifications | 2 (1.0%) | 50% |
| Accommodation | 2 (1.0%) | 50% |
| Accommodation, supported independent living, assistive technology, home modifications | 2 (1.0%) | 100% |
| Accommodation, supported independent living, assistive technology, home modifications, behavioural support | 2 (1.0%) | 100% |
| Assistive technology, behavioural support | 1 (0.5%) | 100% |
| Supported independent living, assistive technology, home modifications, behavioural support | 1 (0.5%) | 100% |
| Accommodation, assistive technology, home modifications, behavioural support | 1 (0.5%) | 100% |
